# Community Heroes and Sleeping Members: Interdependency of the Tenets of Energy Justice

**DOI:** 10.1007/s11948-022-00384-3

**Published:** 2022-09-14

**Authors:** Mandi Astola, Erik Laes, Gunter Bombaerts, Bozena Ryszawska, Magdalena Rozwadowska, Piotr Szymanski, Anja Ruess, Sophie Nyborg, Meiken Hansen

**Affiliations:** 1 Delft University of Technology, Jaffalaan 5, 2628BX Delft, The Netherlands; 2 Eindhoven University of Technology, VITO, Eindhoven, The Netherlands; 3 Wroclaw University of Economics, Wrocław, Poland; 4 Technical University of Munich, Munich, Germany; 5 Technical University of Denmark, Lyngby, Denmark

**Keywords:** Energy cooperatives, Energy justice, Justice issues, Renewable energy

## Abstract

Energy justice literature generally treats its three tenets, distributional justice, procedural justice and recognition justice, as separate and independent issues. These are seen as separate dimensions by which criteria can be formulated for a just state of affairs. And a just state of affairs regarding energy should fulfill all criteria. However, we show, using empirical research on six European energy communities that the tenets of energy justice are interdependent and negotiated in practice. We show this interdependency using three core concerns of justice—risk, effort and power—which we identified through our empirical work. Our findings reveal that community members are often willing to take risks and put in effort, if they are compensated with more power within the community. Similarly, members are willing to compromise power if no effort or risk-taking is required from them. This demonstrates the interdependency of the tenets “procedural justice” and “distributional justice” within energy communities. We reflect on the need for energy justice theory and policymakers to recognize the significance of this interdependency.

## Introduction

Renewable and community-driven energy projects have received much praise for their potential in securing a just energy transition. Recent scholarly work has however taken a more critical approach, using the lens of energy justice to reflect on internal dynamics of local RECs in their interaction with broader local, national and regional decision-making (Mundaca et al., 2018; Roeser & Pesch, 2016; van Bommel & Höffken, 2021). In this work, justice issues within energy cooperatives are analyzed using the framework of three tenets of energy justice. The framework splits justice issues into three types: distributional justice, procedural justice and recognition justice.^1^ The tenets are generally treated as three separate categories. However, existing literature has not yet been explicit about the interconnections of these tenets and how they, together, contribute to a just state of affairs in RECs. How, for instance, is one type of justice weighed against another? And are some tenets more important than others? How do the tenets relate to each other? A theory of justice cannot make judgements on whether a particular state of affairs is just or not without being explicit about the interconnections between the theory’s principles. If the interconnections between the tenets of energy justice were made clearer, then the framework could be more normative about what kinds of RECs are just and which aren't. Our empirical research provides some insights regarding the connections between the tenets of energy justice in RECs.

The aim of our research was to investigate, in an open and explorative manner, energy justice issues within the context of RECs in different European countries. We decided to research this question through a qualitative approach, reflecting on when RECs were seen as satisfactory or unsatisfactory by members of RECs. We used a grounded theory methodology to study six European renewable energy communities.

We define RECs as: communities of people connected through formal or informal membership in a project or initiative which seeks to provide the members with renewable energy. We intentionally chose to investigate energy communities broadly, rather than sticking to the legal definition because the we wanted to include an REC in-the-making for maximum variance, which does not yet fit the legal definition.^2^ Our explorative research into energy justice issues in RECs led us to insights that could benefit energy justice theory as well as policy and governance regarding RECs.

Energy justice more broadly treats its three tenets as separate issues, each being essential for energy justice. Our research however led to findings that call this idea into question, in the case of energy communities.

## Energy Justice and REC’s

Given the environmental and social costs of energy production, energy technology has become an ethically and politically charged topic. In response to this, the field of energy justice research has emerged. The field investigates justice issues that arise in connection to the production, distribution and use of energy (Heffron et al., 2015; K. E. H. Jenkins et al., 2021; K. Jenkins et al., 2016; Miller et al., 2013; B. Sovacool & Dworkin, 2014; B. K. Sovacool et al., 2016). The dominant approach in energy justice literature was first proposed by McCauley et al., 2013 and developed further by others (K. Jenkins et al., 2016; Mccauley, Heffron, & Jenkins, 2013; B. K. Sovacool & Dworkin, 2015). This approach refers to three tenets of energy justice:

- *Distributional justice*
- *Procedural justice*
- *Recognition justice*.

Distributional justice is geared at just outcomes in terms of the distribution of benefits and disadvantages (K. Jenkins et al., 2016). Distributional justice aligns with various philosophical ideas about the importance of distributing goods and bads in a just society.

The second tenet, procedural justice, is the sense of justice which is concerned with the justice of the process by which benefits and disadvantages are distributed. It is concerned with how decisions are made and whether the procedures to make decisions are just (K. Jenkins et al., 2016). Generally, energy justice literature favors democratic processes as being more procedurally just (Mccauley et al., 2013; B. Sovacool & Dworkin, 2014; Stephens, 2019; Stephens et al., 2018; Szulecki, 2018; Van Veelen, 2018).

Recognition justice places importance on the inclusion of people, especially marginalized groups. An example of a recognition justice issue would be a failure to recognize the energy needs of people, some of whom, for instance, depend on a higher indoor temperature for health reasons (K. Jenkins et al., 2016). Sometimes recognition justice is seen as falling under procedural justice.

According to some, RECs are a promising way to bring about a just energy transition (McCabe et al., 2018). Many energy communities also take various initiatives to make sustainable energy more accessible to underrepresented groups (Hanke, Guyet, & Feenstra, 2021). One could say that RECs redistribute power over the energy market to citizens. However, recent literature has also been critical.

For example, RECs redistribute risks of the energy transition to individual participants or households (Adams & Bell, 2015). More democracy is generally praised as making the energy system more just. However, in reality inequalities of power can exist within RECs which are not immediately visible in the formal structure of the REC (Van Veelen, 2018). Mundaca et al. describe cases of RECs where the procedures of decision-making are fair, but the end-result tends to benefit some people more than others (Mundaca et al., 2018). Localist energy policy has also been criticized for being unrealistic, deferring responsibility from the state on to unwilling citizens and making unfounded assumptions about local scale being more democratic (Catney et al., 2014; Purcell, 2006). Other literature speaks about the green energy transition including a sacrifice of communities who are disadvantaged by green energy technologies (Finley-Brook & Thomas, 2011; Scott & Smith, 2018). The critical literature on localism and inequality in RECs hints at the idea that RECs could become, in the worst case, “sacrifice zones” who bear a larger than average share of the risks of the energy transition. This highlights the urgency of understanding what makes an REC just.

Literature reviews show that energy justice literature tends to use the *three tenets of energy justice*, although work using other approaches exists (Heldeweg & Séverine Saintier, 2020; Lacey-Barnacle, 2020; Rasch & Köhne, 2017; van Bommel & Höffken, 2021; van der Horst, 2014). While literature on the three tenets of energy justice and RECs exists, there is little engagement with the question of whether some tenets are more important than others and what the minimal conditions for justice are. For instance, Mundaca et al. argue that their cases scored well on procedural justice and less well on distributive justice (Mundaca et al., 2018). Van Veelen makes a similar point, that accounting for the tenet of procedural justice with local control and autonomy can sacrifice other areas of justice like equality (Van Veelen, 2018). It has also been shown that distributive and procedural justice are important in determining amount of citizen support for energy projects (Walker & Baxter, 2017a). No conclusions are drawn about whether REC cases as a whole are just or not. The tenets of justice are often treated as separate aspects which contribute to justice. Van Bommel and Höffken approach energy justice in RECs in this way too (van Bommel & Höffken, 2021). The authors highlight some procedural, distributive and recognition justice issues within RECs from literature, but remain neutral on their interconnections and the respective normative weight of each issue. Literature remains agnostic about how much democracy or equality is enough and whether it is necessary for the fulfillment of all tenets.

Philosophical theories of justice often contain multiple principles, just like the energy justice framework. However, generally a ranking of the principles and a description of their interrelations is given. For example, a crucial part of Rawls’s theory of justice, in addition to the principles, is a prioritization of the principles. The principle of fair equality of opportunity comes before the difference principle: meaning that it is more important that inequalities are attached to offices open to all, than that the inequalities are to the benefit of the least advantaged. These two principles make up the second principle of justice, which is lower in priority than the first principle (Rawls, 1971). This kind of scheme does not exist for the energy justice framework, making it difficult to understand when exactly an REC can be deemed just. Creating such a scheme, where the tenets of energy justice are related to each other, requires understanding the interrelations between the tenets in RECs. Our findings on these interrelations emerged from an empirical study of RECs.

## Selection, Method and Description of Case Studies

### Selection and Method

We collected qualitative data from 6 energy communities. These were located in Belgium, the Netherlands, Poland, Denmark and Germany. We approached the cases and set up interviews with members. We conducted semi-structured interviews with members, asking about the energy community they were involved in, what they think about it and what problems and benefits they experience. We collected from each case study distinctive justice issues. Through analyzing interviews and hosting workshops and sharing case study narratives, we were able to identify core concerns in the cases and patterns in the way those core concerns manifested.

Three basic REC characteristics were used to compile a pragmatic set of European case studies to analyze with optimized representativeness and validity how the tenets of energy justice emerge in RECs. We adopted a purposeful sampling method: we identified cases by relevance to topic of interest (Creswell, 2006). We used maximum variation sampling: we selected cases which are as diverse as possible with regard to the topic of interest in order to document a broad range of dimensions to a topic (Creswell, 2006). In our case the topic of interest was justice and energy community governance.

We selected our cases, for maximum variation, from European countries with a strong energy community movement or tradition like the Netherlands, Germany and Denmark (Beuse & Organisationen for Vedvarende Energi, 2000; Forsman et al., 2020; Holstenkamp & Stier, 2018; Karnøe & Jensen, 2016; Nyborg & Røpke, 2015) in contrast to countries with a weak or emerging energy community movement or tradition, such as Poland (Adamczyk & Knyszewska, 2017; Producent, Energia, & Energia, 2012) with Belgium being somewhere in between. We also selected some RECs aimed at low income residents and some aimed at high income residents. Some of our cases experiment with cutting edge technological developments (e.g. virtual power plant like in the Belgian case, peer to peer trading in the Danish case) in contrast to cases that rely on more traditional technology and business models (like the German case, with its traditional cooperative structure and renewable energy sources). Most cases were cooperatives which were already running, but we selected one (Brainport Smart District) which is in the planning-phase, in order to observe the setting-up of a cooperative.

Each case was studied and analyzed by a researcher in the team. We collected from each case issues which participants of the REC were dissatisfied about. The dissatisfaction or satisfaction of participants was assessed by the researcher who spoke to them. We collected points of dissatisfaction, which clustered into 3 themes. We labeled these themes core concerns. The core concerns, we discovered, were linked to each other and together explained the dissatisfaction or satisfaction of participants.

Table 1 shows the six selected cases and an overview of the empirical methods used. Central and overarching in all cases were the participant interviews to enable comparison, complemented with specific methods based on contextual boundary conditions to collect empirical data.

**Table 1:** Empirical material for each case study

Energy community	Empirical methods
Housing Cooperative South in Wroclaw	11 interviews, survey research, panel, workshops, participatory observation
Brainport Smart District in Eindhoven	Interviews with 9 participants, observations at meetings and public events, participatory observation
Nautilus in Amsterdam	Interviews with 3 individual participants and one group interview with 3 participants simultaneously. Observation at one meeting
Erneuerbare Energien Rottenburg (eER)	Interviews with 3 individual participants, analysis of 261 documents reduced to 24 core documents, participatory observation
EnerGent cooperative (Flanders region, Belgium)	6 interviews, meetings with researcher doing case study research on EnerGent in the context of the Interreg cVPP project
The Energy Collective/Svalin (DK)	7 qualitative interviews with participating researchers, 1 group interview with 3 inhabitants, participatory observations in the community

### Housing Cooperative in Wroclaw (Poland)

The Housing Cooperative Wroclaw in Poland, henceforth called HC Wroclaw was initiated to decrease the costs of living and carbon emissions. The project covers 102 buildings and nearly 11,000 dwellings, in which about 30,000 inhabitants live. The first stage of the project consisted of investment in a solar power plant for the highest buildings. The second will involve producing energy with photovoltaics for running air source heat pumps. The solar power plant was developed in consultation with inhabitants.

### Brainport Smart District in Helmond (the Netherlands)

Brainport Smart District is a cooperation between Eindhoven University of Technology, the municipality of Helmond and various other organizations to build a livable, healthy, energy-neutral and sustainable district which serves as a testbed for new technologies. One of the design principles of the project prescribes renewable energy generation in the district. The first inhabitants have been invited to take part in designing the part of the district where they will live. The future inhabitants must also collectively make decisions about how and whether they will produce their own energy. The infrastructure of the district is still being planned simultaneously. Hence, the residents are furthering their own plans, occasionally with the help of BSD organization, within a risky and changing context. The process is still ongoing at the time of writing this article (2021).

### Nautilus in Amsterdam (the Netherlands)

Nautilus is a housing cooperative in Amsterdam that built their own housing complex co-creatively. Getting permission to install a heat pump with photovoltaics required a legal battle. Now inhabitants have been living in the complex for 3 years. They run the complex and the maintenance of the heat-pump through voluntary initiatives. The residents have extensive democratic procedures for decision-making which diverge from legally defined cooperative voting procedures. Each resident has veto power.

### Erneuerbare Energien Rottenburg (Germany)

Founded in 2009, Erneuerbare Energien Rottenburg eG (eER) aims at advancing regional small-scale renewable energy projects as well as shaping the German energy transition from the grassroots, mainly through solar PV and wind power projects. eER currently counts more than 200 members. Financing is provided by the members’ shares, external financing and internally generated capital. The community sells the electricity generated to the property owners who provide their rooftops to eER, mostly the city of Rottenburg. Their individual consumption is turned quasi-renewable, as the electricity generated in eER equals the approximate amount of electricity consumed by the total of its members.

### EnerGent in Gent (Belgium)

The EnerGent cooperative originated from social initiatives organized by volunteers in the Macharius neighborhood, which is a disadvantaged neighborhood in the city of Ghent in Belgium. Around 2013, the idea arose to invest in renewable energy in Ghent through a citizen cooperative. The EnerGent energy cooperative was founded as a place-based community driven by the goal of sustainability, but also to reduce inequality by making energy generation more accessible for low-income households (van Summeren et al., 2020). For the time being, they still account for around 50% of their income from subsidies, but the intention is to reduce this percentage in the future.

### The Energy Collective/Svalin (Denmark)

The Energy Collective is a “living lab” project that involves the housing cooperative Svalin, near Roskilde in Denmark. The focus is mainly to develop ways to share or trade energy among people living in communities – in other words, they are looking at “new approaches for all to directly exchange energy in a truly consumer-centric manner” (The Energy Collective, 2021). Blockchain is one of the main innovations that is being explored. The project website reads: “Houses and shared infrastructure there were designed to accommodate solar panels, a geothermal heat pump and electric vehicles. The community as a whole is energy positive, meaning that it produces more renewable energy than it consumes, on a yearly basis.” (The Energy Collective, 2021).

## Findings: Risk, Effort and Power as Core Concerns Within RECs

Three *core concerns* emerged from our interviews and observations. The core concerns are what justice issues in RECs are generally about: risk, effort and power.

We understand risk as the chance of negative consequences for a participant, such as financial loss, property damage or troubles with other people that can happen as a result of entering an REC. We understand effort as the time and work that participants put into maintaining or improving the REC. We understand power as the ability of participants to influence affairs and decisions within the REC.

We observed that whether the justice issues were seen as problematic or not depended on the balance of the impact of these core concerns of the participants. There seemed to be two justice equilibria, in which the state of affairs was considered just. The first equilibrium is a state where participants are exposed to low risk, with low effort required from them, whilst having a low amount of power in the REC. The second justice equilibrium is where participants are exposed to a high risk, with a lot of effort required from them and have a high amount of power in the REC. We will now describe how these just equilibria were manifested in our cases, as well as how unjust states of affairs emerged. We will describe three states of affairs which were experienced as just by members, with regard to the internal functioning of the REC. We exclude concerns of justice regarding issues external to the REC, like whether participants felt their REC was treated justly by legislation of the country they were in.

### Community Heroes: High Risk, High Effort, High Power

Some RECs consisted of very active and risk-taking members. One of our cases, Nautilus in Amsterdam, had initially involved a very high risk for participants. In the planning phase, when the group had been established in order to design, plan and finance the building of the house, each member contributed 16,000 euros, with a risk of losing the money. As time passed and more of the money was spent, the chance of getting the money back decreased. Some inhabitants described the stress that was caused by various disappointments, like the social housing corporation which pulled out of the project or the miscalculation of costs leading to a sudden need to cut 2 million. For Nautilus, the heating installation posed a large financial and technical risk. The risk materialized after the construction when many defects were discovered, including a large leak. Having the leak fixed required, according to the inhabitants at the time of the interviews, juridical action against the contractor.

The housing complex and the heating system took a lot of effort to build and still takes a lot of effort to maintain. Various mistakes of the contractor in building were found afterwards and took a long time to get fixed. Some inhabitants expressed a desire to carry out less house-related duties.

Although participants took great efforts and high risk, what they got in return was a substantial amount of power within the cooperative. In Nautilus we saw the justice equilibrium *high risk high effort high power*. Nautilus works with a decision making process, designed by a member, based on consensus which does not conform to the legal standard of voting in cooperatives. Every person in the cooperative has a veto power on all decisions. This means that one person can block any decision, and if that happens then the majority must propose an acceptable alternative to the disagreeing person. The interviewees viewed this system as being more democratic than voting. One of them mentioned that the reason they had to put an emphasis on democracy was the sensitivity of the financing of the project, especially at the beginning. This narrative portrays the sentiment that if everyone takes a big risk, then they must be given enough say about how things are done. This supports the idea that RECs sometimes compensate for risk with decision-making power.

Some of our case studies were “living lab” projects, which meant that the project was aimed at experimenting with new technologies. In the Svalin community in Denmark and Brainport Smart District in Eindhoven, the innovativeness of the technology and the risk it caused was also met with a movement towards extensive consent-driven procedures. Voting was considered too limiting. BSD and Svalin also had extensive discussions on how the decision making structure should be.

In Svalin this discussion was prompted by an event. A vote was held on whether the communal dinners should be vegetarian. The vote resulted in a decision for allowing meat, which led to a very active household leaving the community. After this, the community began working with other ways of voting, instead of relying on for and against votes, which were considered too limited. While debating power structures explicitly among members can be considered more empowering than sticking to an uncontested power structure, it is not always practical especially in large communities. The case studies that did this were all smaller in size.

### Sleeping Members: Low Risk, Low Effort, Low Power

Other cases consisted of less active and less risk-taking members. We found that some cases imposed a low risk on participants, required low effort and granted participants low power. This was not felt to be unjust by participants. It is alright to have little influence in something which imposes little risk on you and requires little effort.

HC Wroclaw adopts a minimally democratic but easy strategy because of a general adversity to change among residents and employees. All projects are carefully prepared by the project team to avoid resistance. Any risks in the project and sources of possible resistance are identified. The practice shows that a relatively small number of active and organized members can effectively implement changes by building a rhetorical advantage. The vast majority, therefore, is not granted much power in the process, other than a vote.

In HC Wroclaw and EnerGent, which are big communities, decision-making structure is set and stuck to according to legal default. This default consists of a board and the possibility for members to vote on issues. Power is centralized and this structure is not contested. In EnerGent, there is a formal democracy as each member has in principle one vote per issue, and there is a general assembly once a year. However, the agenda is set by a limited group of members who, because they possess specific resources (e.g. technical, juridical knowledge) represent the community as a whole and act on their behalf. In the case of eER, the formal hierarchy also follows fixed statutes: the board is appointed (for three years with the possibility of re-appointment) and recalled by the supervisory board. The supervisory board is elected by the general assembly for three years with the possibility of re-election.

However, the residents in eER, EnerGent and HC Wroclaw did not generally find it problematic that their democratic control was minimal. This is because not much risk was taken by the majority of participants and not much effort was required. Therefore, there was not much at stake for most participants. HC Wroclaw had removed most of the risk and burden to members. HC Wroclaw used a pilot project to eliminate some technology-related risks. A pilot project led to the discovery of a flaw in the attachments of photovoltaics, which would have caused the roof to leak. The project was also financially de-risked due to an external funding partner. The governance structure was traditional and legally supported. Participation in EnerGent is to a large extent de-risked. 60% of investments are covered by the project funds. The project involved only tests under simulated circumstances (e.g. simulated dynamic prices) so there was no substantial risk for the households. The project also targeted low income participants and used a similar strategy as HC Wroclaw. The low amount of power for participants, in these cases, is compensated by the lack of risk and lack of burden for, making the situation acceptable to our interviewees.

The dynamic of power compensating for risk and effort can also be observed at the level of individual decisions on how much to participate in the REC. HC Wroclaw, EnerGent and eER all involved community-hero residents who participated in the board, and a host of sleeping members, who participated less. Those who took functions as board members were often accepting of the fact that other members did not put in as much work as they did. The board members, individually, had more power, in return for their effort. The fact that most members did not participate actively was not seen as a problem in these cases. As one of the block leaders in HC Wroclaw commented “it is natural for such a sizable organization that most of the persons do not get involved proactively in the community life.” eER’s board consisted of only three members out of a total of 230 members. The board members stated that their commitments amount to a lot of work, but they do not perceive this distribution as unfair. They perceive their individual workload a contribution to Germany’s energy transition.

### Unjust State of Affairs: High Risk, High Effort, Low Power

When risk, effort and power were not well balanced, participants in our cases experienced unfairness. If one takes a large risk and puts a lot of effort into an REC, but still has a low level of influence, one may feel wronged. Intuitively, it seems like this is justifiable.^3^

An example of this occurred within Brainport Smart District. In the project, which is participative and co-creation-focused regarding other areas than energy too, the risk for the participants was framed as a part of their conscious choice to “pioneer” in “the smartest district in the world.” Many of the participants were comfortable taking risks, as long as they would get to choose how their district would look.

Initially, participants believed to be part of a high risk, high effort, high power project. This justice equilibrium only fell apart at the moment that a lack of clarity and information within the BSD project as a whole constrained the future residents’ ability to plan and take action. The multitude of other parties and their indecision made the future residents structurally powerless. At this point, the system became a *high risk high effort low power* -system, which was perceived as unjust by participants.

Individuals within RECs can also experience an individual lack of due power for the risk one takes and the effort one puts in. A resident of Nautilus expressed sympathy for the idea that those who put in effort should have more influence. He felt that the extensive democratic procedures, which were in place to increase each members power, were in some sense failing the people who were putting in effort, or taking initiative. “I am used to it being so that the one with the paint bucket decides what color the walls are, that initiatives are encouraged. And that there is a lack of initiatives, so the people who take initiatives get the freedom to do it the way they want. But here that is completely turned around… …What happens now is that people come in with an initiative, and then there are always 2 or 4 people who say: we’re not doing that. And that changes the initiative… …And there is a meeting and eventually your nice initiative turns to some grey mash… … There is so much resistance against everything” This highlights a possible perspective where equally distributed power is felt as unjust since some members put in more effort than others.

Individuals could end-up in an unjust situation also due to being exposed to a larger risk than other participants. In Brainport Smart District differing levels of commitment among the future residents caused an unequal distribution of risk. A participant stepping out of the project would mean a re-planning for everyone. If one person steps out, there is one household less, which requires spatial and energy-related re-organization, costing time and possibly money for participants. Some of the very committed members perceived the lack of commitment from others as a risk for themselves.

Different people have different needs for becoming empowered. One participant at BSD worried that the wishes of certain people were being ignored, because those people are shyer or less loud in meetings. An interviewee from Nautilus echoed a similar worry “If two people voice an opinion very passionately, then they often get what they want.”

Apart from differences in sociability or rhetorical skill, difference in knowledge and know-how were also mentioned as factors that determine the level of empowerment. According to the statutes of eER, any member of the community can be elected supervisory board member or appointed board member. However, in practice, executive roles require expertise in business, administration and energy technology. Because of this, some participants found it difficult to participate in the community in these circumstances. BSD participants expressed similar worries. This goes to show that some members in communities can have needs regarding type of dialogue, use of terminology and atmosphere, which are not always recognized by others. Personal differences, therefore, can sometimes limit the power of individuals within an REC, causing some members to lack due power individually.

## Conclusion

### Conclusion for Theory: Energy Justice Theory Should Expand to Include the Interdependency of Tenets

Philosophical theories of justice, like that of Rawls (1971), provide principles of justice as well as a prioritization of principles. Because the energy justice framework does not yet provide such a prioritization, scholars have only been able to point out justice issues in RECs but not determine when an REC is just or unjust all things considered. The importance of a balance between risk, effort and power allows us to create a more comprehensive framework.

Risk and effort are matters of distributive justice or how the costs and benefits are distributed. Questions of power could be classified as a matter of procedural justice, or how decisions are made and who gets to influence them. Therefore, the fact that risk and effort can be compensated with power means that distributional justice issues can be compensated with procedural justice. One tenet of energy justice can, in practice, compensate for another enough to make the situation satisfactory to members. Similarly, a lower amount of power, an issue of procedural justice, can be compensated with less risk and less effort. Less risk and effort can be seen as a distributional benefit as well as an increase in recognition justice, because it allows those with low incomes or those that are more risk averse to join the REC.

This has important theoretical implications, regarding the link between the different tenets. It means that the tenets of justice are interdependent. Actions that procedural justice requires depend on the distributional aspects of the REC. What can be considered a just amount of power depends on how much risk and effort one puts in. And that the actions that are required by distributional justice depend on procedural matters. A just amount of risk and burden is dependent on how much influence one has. Secondly, the negotiation between risk, effort and power can also be seen as a tradeoff between distributional, procedural and recognition justice.

Work on community energy treats the tenets of energy justice as separate components of a justice framework (Frate, Brannstrom, de Morais, & Caldeira-Pires, 2019; Gross, 2007; Liebe et al., 2017; Mundaca et al., 2018; Walker & Baxter, 2017b). However, as these findings show, there is a need to treat them as interdependent, meaning that no conclusions can be drawn about one, without drawing conclusions on the others.

### Conclusion for Practice: REC Governance and Policy Can Support the Negotiation of tenets

Energy communities internally are clearly not immune to justice concerns, despite their empowering potential. The justice equilibria and unjust states that we found among our cases highlight the centrality of risk-bearing and effort in renewable energy communities and how risk-bearing affect the internal politics and ethics of these communities. The negotiability of risk and effort with power means that anyone engaging in REC governance must acknowledge and quantify each core concern and determine whether they are in balance. REC governance is influenced by policy and therefore our findings are relevant for energy policy too.

The choice between the justice equilibria could even be seen at the level of individual members. Members in communities sometimes choose to be ‘community heroes’, taking up *high risk, high effort, high power* roles, joining the board or starting or participating in initiatives. Members also sometimes choose a *low risk, low effort, low power* role, being one of the less active members in the community.

This choice appears to individuals joining an REC and for policy makers. An REC member must decide which role to take and may ask themselves the question: Which is more valuable to me, influence or freedom from burden? And from the community-perspective, one may ask, in which role am I more advantageous for my community, as a community-hero with lots of burden and influence, or as a sleeping member with little of both? Both of these roles have their respective moral demands. The amount of community-heroes and sleeping members that an energy community needs to function depends on the context and the legislation surrounding energy communities.

For the policy maker, the dilemma revolves around questions regarding community heroes and sleeping members: In whose benefit should REC policies be? Should they benefit those who take on burdens and risks, or those who do not, and perhaps cannot, do so? Should REC-related policies stimulate citizens to become community-heroes, or is this unfair to those who cannot? Are benefits for sleeping members perhaps unfair to community-heroes?

A practical example of how the justice equilibria interact with policy: While legally defined default structures served well for some *low risk, low effort, low power* communities, they were considered too limiting by the *high risk, high effort, high power* communities. This goes to show that policymakers should be aware of the different justice equilibria among energy communities, for policy to be fitting.

In this paper we have shown our case studies to reveal that the tenets of energy justice are in practice negotiable and interrelated. Of course, our case studies are only six and more research would be needed in order to draw conclusions about general trends among RECs. The results of this study should be treated as a starting point for philosophical analysis regarding the three tenets of energy justice. These findings show that it is at least thinkable that the three tenets are interrelated and negotiable rather than separate ideals.

This raises various normative questions about whether this interrelation is ultimately justified according to moral theory, and whether it is justified when taking into account various external factors affecting the energy market. For instance, even if groups of community heroes, or high risk, high effort and high power groups are satisfied with their REC, is it fair that citizens take upon themselves the risks of the energy transition like this? Further normative research is needed on whether such high-risk RECs are justified in the greater scheme of things.

## Data Availability

Our interviewees gave consent for the collection of data and its use in publications.

## Footnotes

1 See Mccauley et al., (2013) for tripartite framework of energy justice.

2 See (Roberts, Frieden, Research, Stanislas D’herbemont, & Eu), 2019) for definition of RECs (renewable energy community) in EU legislation.

3 We tend to think that effort, especially useful effort which benefits others, should be rewarded with esteem or respect, especially in a friendly, familiar or communal setting. Esteem and respect for a person are often expressed by listening to and taking seriously the interests of that person. At the same time, it might also be seen as undemocratic to grant one individual more power just because they put in more effort for the community. This article focuses on the negotiation that seems to happen in RECs, however, the normative question “Does someone putting in effort merit granting someone more power in an REC?” merits further philosophical investigation.

## Declarations

### Human and Animal Participants

Interview transcripts are not made public. This is done to protect privacy of participants.
