# Correction: Community Heroes and Sleeping Members: Interdependency of the Tenets of Energy Justice

**DOI:** 10.1007/s11948-022-00413-1

**Published:** 2022-11-02

**Authors:** Mandi Astola, Erik Laes, Gunter Bombaerts, Bozena Ryszawska, Magdalena Rozwadowska, Piotr Szymanski, Anja Ruess, Sophie Nyborg, Meiken Hansen

**Affiliations:** 1 Delft University of Technology, Jaffalaan 5, 2628BX Delft, The Netherlands; 2 Eindhoven University of Technology, VITO, Eindhoven, The Netherlands; 3 Wroclaw University of Economics, Wrocław, Poland; 4 Technical University of Munich, Munich, Germany; 5 Technical University of Denmark, Lyngby, Denmark

## Correction to: Science and Engineering Ethics (2022) 28:45 10.1007/s11948-022-00384-3

In this article Gunter Bombaerts was incorrectly linked to 'Delft University of Technology, Jafalaan 5, 2628BX Delft, The Netherlands', due to an incomplete correction. He should have been linked to 'Eindhoven University of Technology, VITO, Eindhoven, The Netherlands', as he was in the original manuscript.

The original article has been corrected.

